# Enabling participation for disabled young people: study protocol

**DOI:** 10.1186/s12889-018-5652-x

**Published:** 2018-06-08

**Authors:** Penelope Carroll, Karen Witten, Octavia Calder-Dawe, Melody Smith, Robin Kearns, Lanuola Asiasiga, Judy Lin, Nicola Kayes, Suzanne Mavoa

**Affiliations:** 1 SHORE & Whariki Research Centre, Massey University, Auckland, New Zealand; 2 School of Nursing, University of Auckland, Auckland, New Zealand; 3 School of Environment, Univeristy of Auckand, Auckland, New Zealand; 4 Centre for Person Centred Research, Auckland University of Technology, Auckland, New Zealand; 5 Melbourne School of Population & Global Health, University of Melbourne, Melbourne, Australia

**Keywords:** Disability, Young people, Community participation, Inclusion/exclusion, Wellbeing

## Abstract

**Background:**

Participation in community life is vital for health and wellbeing, promoting a sense of belonging, networks of social support and opportunities for physical activity. Disabled young people have lower levels of mobility and participation in recreational activities (physical, social and cultural), education and employment, than their peers without disabilities. This has implications for their health and wellbeing and life course opportunities. Previous research on the participation levels of disabled young people has primarily relied on parent/caregiver reports and been oriented to home and school environments. This study investigates how physical and social environmental factors cohere to support or restrict the everyday mobility and participation of disabled young people.

**Methods/design:**

The study is located in Auckland, Aotearoa/New Zealand (NZ). Participants comprise 35 young people aged 12–25 years with mobility, vision or hearing impairments. A mixed-methods research design combines objective (global positioning systems, accelerometers, geographical information systems) and self-report measures (travel diaries, and questionnaires) to assess young people’s mobility and levels of participation in leisure/educational and employment activities with in-depth interviews exploring their everyday experiences of inclusion/exclusion, and factors enabling or constraining community participation. Parents/caregivers and disability sector key informant viewpoints on the community participation of disabled young people have also been gathered through in-depth interviews. Follow-up workshops with young people and parents/caregivers will identify pathways to increase participation and challenge current disabling practices.

**Discussion:**

This study looks beyond barriers in the physical environment to the interplay of personal, social and physical factors that enable or constrain the community participation of disabled young people. In keeping with the study’s overarching goal of increasing opportunities for effective community participation and full citizenship of disabled young people, research methods were applied flexibily – negotiated and adapted to maximise each young person’s participation in light of their abilities and preferences.

## Background

Community participation – engagement in the social, economic and cultural life of a community – is crucial for the health and wellbeing of young people [1, 2]. It promotes physical and mental wellbeing through pathways such as a sense of belonging, opportunities for physical activity, and networks of social support. In Aotearoa/New Zealand (NZ) as elsewhere, evidence indicates substantial inequalities in community participation – and health and wellbeing outcomes – for disabled young people compared with non-disabled peers [1, 3, 4]. Disabled young people have lower rates of participation in sport and recreation, post-secondary education, training and employment (and as adults, an unemployment rate 50% higher than for those without disabilities in NZ [5]).

The NZ Disability strategy defines disability as a process which happens “when one group of people create barriers by designing a world…taking no account of the impairments of others” (p.3) [6]. Barriers to participation in the public realm and community life for disabled young people exist across a number of domains. In the built environment domain, barriers include poor access to transport and amenities as well as streets and other public spaces that can be difficult to negotiate [7, 8]. Limited educational and employment opportunities can create barriers to participation in economic, social and cultural domains. Further, the taken-for-granted acceptance of able-bodied privilege sustains discriminatory discourses, and the lack of recognition of the value and rights of disabled young people. The pervasiveness of ‘ableist’ assumptions and practices is particularly damaging as these can undermine individual agency and collective attempts to reduce other barriers [9–12].

Studies examining environmental determinants of participation for disabled young people [9] have largely been conducted with younger age groups, and concentrated on experiences at home and school [13]. Studies have also tended to rely on the perceptions of parents/caregivers, rather than the perceptions of the disabled young people themselves. An observation made by researchers who are working with disabled young people is that data collection is time consuming and requires flexibility of research process and methods [14]. In this field very few studies have investigated the life worlds of disabled young people in the wider community or given voice to their needs in an urban planning context [15, 16].

The importance of looking beyond environmental barriers to the interplay of personal, cultural, and community factors that enable or constrain community participation has been noted [1]. This study engages with disabled young people living with mobility, vision, or hearing impairments to understand their perceptions of and experiences in the public realm; to investigate barriers and enablers of participation; and to explore strategies to pursue change in community structures, social practices and spaces that limit their full participation in NZ society.

### Context

The origin of this study was a disability sector critique on the absence of disabled children in a prior study undertaken by the authors. *Kids in the City* [17] investigated the independent mobility, physical activity and neighbourhood perceptions of Auckland children aged 9–13 years, providing a rich understanding of able-bodied children’s use and experience of their urban neighbourhoods, and the physical and social characteristics which have encouraged or restricted their mobility and community participation [18–21]. In 2014, representatives of the disability sector asked the research team to carry out similar research with disabled young people.

Data collection methods used in our earlier study [17], were trialled in a small pilot with mobility-impaired school-aged participants. Thereafter a proposal was developed (based on recruiting disabled young people aged 12–18 years) and funding secured in 2015. An advisory group of disabled young people was established. In light of the often restricted community participation of disabled children and young people, the group recommended that extending the age range to 25 years would render a richer understanding of factors which could encourage or restrict participation in the public realm. This advice was taken up, the protocol adapted and ethical approval sought accordingly.

### Study aims and objectives

The overarching aim of the study is to foreground the voices of disabled young people to identify and promote pathways for environmental change in order to increase opportunities for their effective community participation and full citizenship.

Specific objectives:

- To explore disabled young people’s community participation and mobility using a modified Children’s Assessment of Participation Enjoyment questionnaire (CAPE), trip diaries, Geographic Information Systems (GIS), Global Positioning Systems (GPS), and accelerometers.
- To access disabled young people’s perceptions and experiences of neighbourhood spaces, services and amenities, and opportunities and constraints they face participating in activities in the wider community via qualitative interviews (home-based and in community settings).
- To investigate discourses of inclusion-exclusion in specific settings as disabled young people and parents/caregivers talk about community participation and mobility practices.
- To identify modifiable environmental factors (physical and social) to increase opportunities for mobility and community participation and to create pathways for the lived experience and priorities of disabled young people to inform local planning.

## Methods/design

The study used a mixed-methods ‘tool box’ approach to data collection [14], combining qualitative, spatial and quantitative methods to gather experiential, mobility, physical activity and participation data.

Introducing the methods as a ‘tool box’ foreshadows later discussion on how methods were adapted to facilitate the participation of individuals in this research. Methods were adapted to accommodate different impairments, ages/life stages, and data collection settings, thereby enabling a high level of participation by young people. Not all methods could be, or were, used with all participants. Participants’ preferred communication style was a factor that influenced how some methods were used. New Zealand Sign Language (NZSL) and alternative and augmented communication (AAC) were employed where appropriate.

At the mid-point of data collection the research team conducted a review of data collection methods. Data were examined and consideration given to: the quality and completeness of the data gathered (respectively for vision, hearing and mobility impaired participants); the understanding of disabled young people’s lives generated by the various methods; and the quality and utility of data relative to the burden its collection placed on particpants.

Ethical approval to conduct the research was obtained by the three universities involved in December 2015 (MUHEC 15–044; AUTEC 15–355, UAHPEC). Variations to the ethical approval were obtained when methodological and method changes were made to the study protocol. Key informant interviews with service providers in the disability sector began in February 2016. Participant recruitment and data collection began in May 2016.

In the following sections we provide a detailed overview of recruitment processes and sample characteristics; data collection methods, including a description of and rationale for adaptations made to various methods at the individual and study levels; proposed analyses; discussion and conclusion.

### Recruitment

A staggered recruitment process took place over 20 months. Recruitment began with school-aged disabled young people who were recruited through educational institutions providing specialist services for vision and hearing impaired young people. Mobility impaired younger participants and older participants were recruited through disability service providers and disability sports and cultural clubs. Initial contact with schools, agencies and groups was via a direct approach from the research team. Introductions and networking opportunities in these settings were also facilitated by key informant interviewees and a NZSL interpreter. Recruitment also took place via websites, publicly available blogs and online newsletters. Snowballing recruitment followed through existing study participants and key informant contacts. Age-appropriate and impairment-specific information sheets and consent forms were provided for disabled young people and parents/caregivers. Disability-related terminology in common use varied between the vision, mobility and hearing impaired communities in NZ and information sheets were tailored to reflect these differences.

### Sample

Participants are a purposive sample of 35 young people (15 aged 12–18 years, 20 aged 19–25 years), with varying levels of visual (12 participants), hearing (10 participants), and/or mobility impairment (13 participants), and their parents/caregivers. As a measure of ‘disability’, participants need to fulfil criteria (or have done so in the past) for On-going Resource Scheme (ORS)^1^ funding, to meet their needs in schools.

A wide range of ethnicities are represented in the sample: 14% identify as Māori (indigenous people of NZ); 48% Pakeha (NZ European); 20% Pasifika (from diverse South Pacific Islands); 11% Asian new migrants; and 6% African new migrants. Participant demographics are summarised in Table 1 below.

**Table 1:** Summary of participant demographics

Participant Groups	Age (yrs)	Number	Gender		Ethnicity				
			M	F	Māori	Pākehā	Pasifika+	Asian^a^	African
Blind and VI participants	12–18	2	1	1		2			
	19–25	10	5	5	1	6	3		
Deaf and HI participants	12–18	8	4	4	2	2	1	1	2
	19–25	2	1	1	1			1	
Mobility impaired participants	12–18	4	3	1	1	3			
	19–25	9	3	6		4	3	2	
Totals		35	17	18	5	17	7	4	2

^a^Asian includes participants identifying as Chinese, Korean, Indian and Filipina/o new migrants

+ Pasifika includes participants identifying as Samoan, Tongan, Cook Island and Solomon Island

### Data collection settings

Settings for data collection have varied. They include homes, schools, other specialist educational facilities, disability-specific recreational settings (Wheelchair Basketball, Sailability, Auckland Deaf Club), and diverse community/public settings (such as cafes, Auckland Maritime Museum, Auckland Zoo, trains, boats) and work-places. Common variations saw researchers first meet with younger participants at school or other educational facilities, whereas older participants have been more likely to choose a café or work-place to meet. The number and mode of data collection contacts with participants and their families (face-to-face and by telephone) varied across the sample from 3 contacts to 12, with a median of 8.

### Data collection methods

After initial contact, a short preliminary interview aimed to establish rapport, outline the project and data collection requirements (in conjunction with official information sheets to meet ethical requirments), and identify any adaptations to data collection procedures required to enable the young person’s full participation in the study. Participant consent – and where participants are under 16 years of age – parental consent was also obtained. Table 2 (below) summarises data collection carried out from May 2016–March 2017.

**Table 2:** Summary of data collection by participant group and age cohort

Participant Groups	Age (yrs)	n(group)	Interviews			Trip diary	CAPE & PAC	Accelerometer & GPS	
			In-depth	Go-along	Parent			7 days	1 day
Blind and VI Participants	12–18	2	2	2	2	2	2	2	0
	19–25	10	10	9	8	10	10	5	4
Deaf and HI Participants	12–18	8	8	5	7	8	8	8	0
	19–25	2	2	1	0	2	2	0	2
Mobility impaired participants	12–18	4	4	4	4	4	4	2	2
	19–25	9	9	8	5	8	9	3	5
Totals		35	35	29	26	34	35	20	13

### Quantitative measures: Trip diary, accelerometer, GPS

On day one the trip diary was explained, the accelerometer and GPS fitted, and wearing instructions given. The first 20 participants wore GPS units and accelerometers for up to seven days and filled out a trip diary for seven consecutive days, with researchers meeting them to download or check data each day (excluding the weekend). Following preliminary analyses of the data, GPS and accelerometer data collection protocols were changed, so that data were collected for one day only for the subsequent 15 participants, and not always at the beginning of the overall data collection process. The participants were asked to select the day they wore the equipment, a day they made a trip from home.

### Trip diary

Trip diaries have been used to record destinations, travel mode and accompaniment status. The example below was completed by an older vision-impaired young person.

**Fig. 1:**
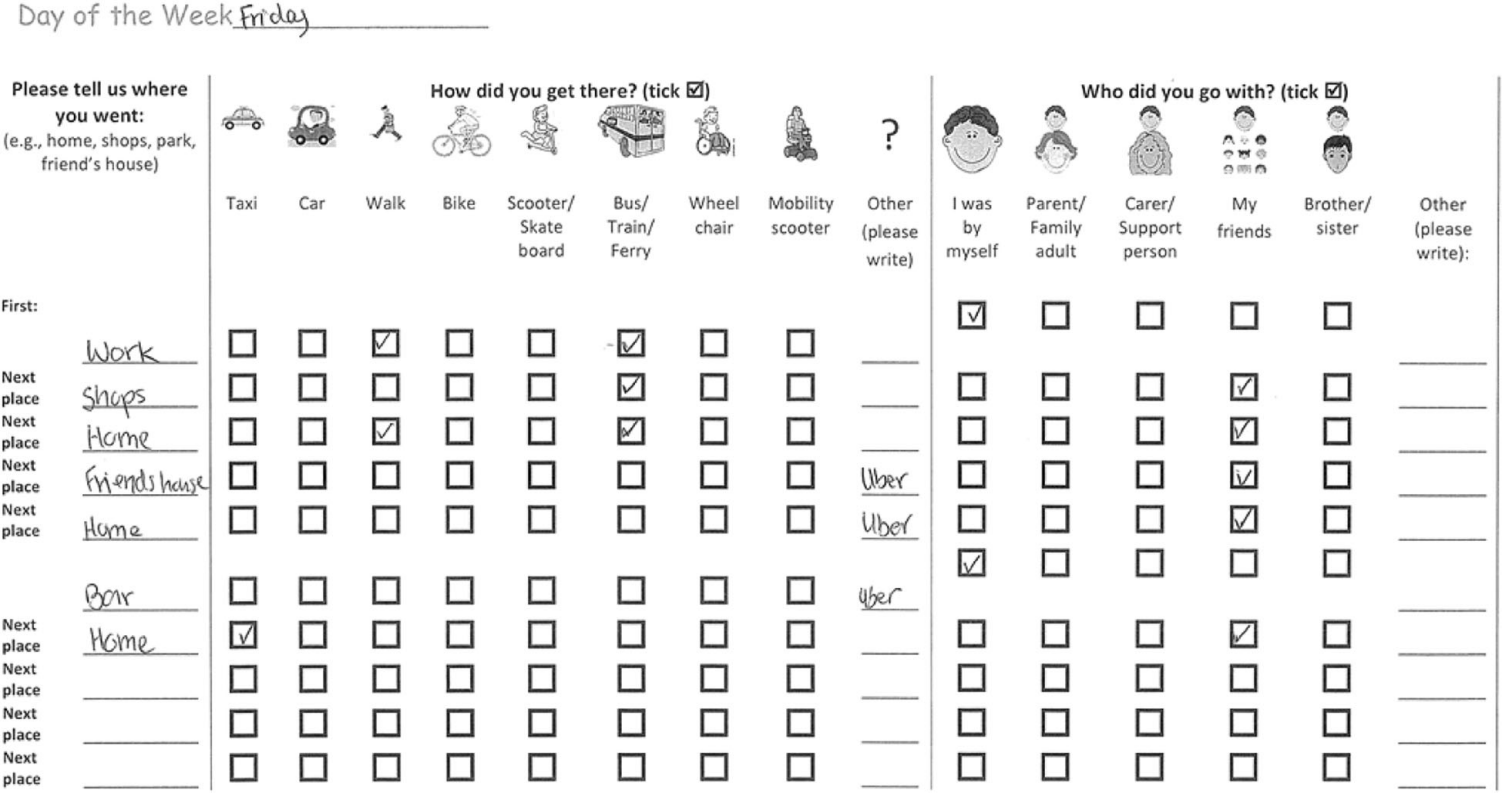
One-day trip diary example

Some participants completed trip diaries themselves, others did so with the help of a researcher, or parent/caregiver, (daily or intermittently) using forms provided, as in Fig. 1 above.

#### Modifications

Variation in data collection methods included vision-impaired participants completing an A3 version of the trip diary (initially sized to A4), deaf, blind and mobility-impaired participants filling out a spreadsheet online version of the form digitally and emailing it to the research team; and mobility-impaired participants talking through their trips with a researcher over the telephone. Initial trip diary forms requested information on trip times and on times of GPS and accelerometer wear. When data were reviewed, time data was frequently incomplete and its accuracy uncertain. Keeping track of time often appeared challenging, particularly for vision impaired young people. Temporal information was not collected for the final 15 particpants recruited. Foregoing this requirement decreased the burden on participants.

### Accelerometer

ActiGraph accelerometers objectively assessed habitual physical activity (PA) over 7 days for the first 20 participants and over one day for the subsequent 15 participants. A 7-day monitoring period is considered reliable in able bodied children [22]; a shorter monitoring period may be sufficient in persons with disabilities due to limited variability in PA across days and floor effects [23].

#### Modifications

**Fig. 2:**
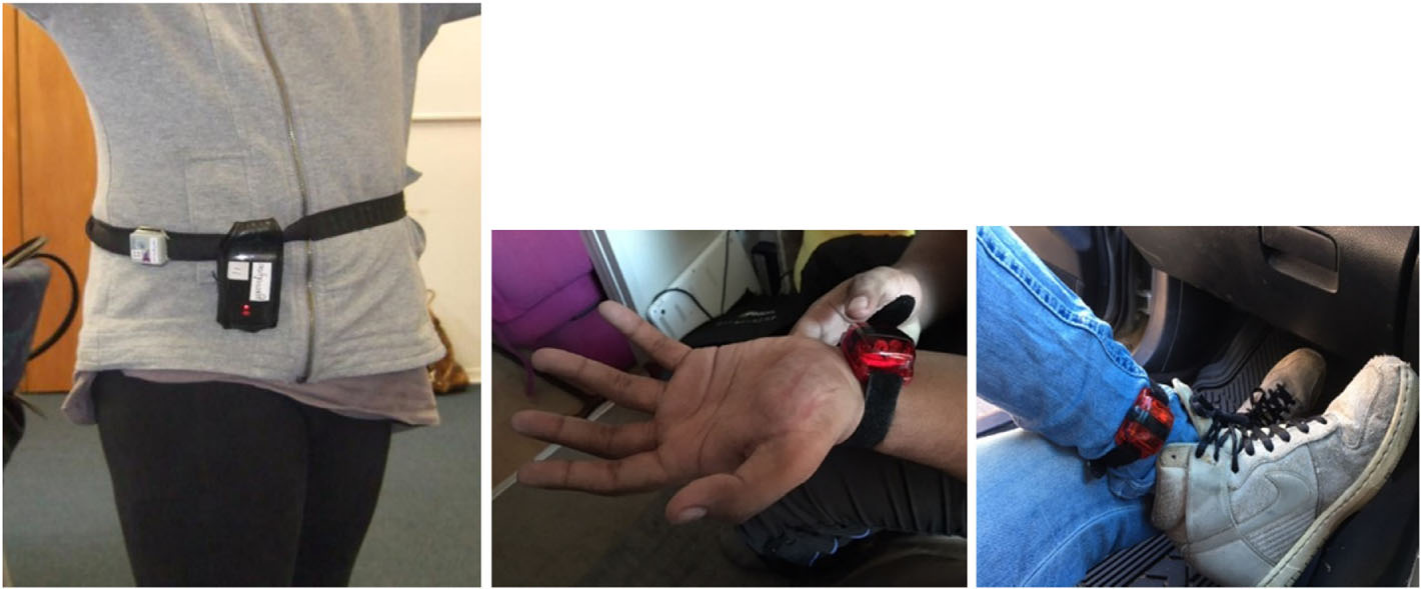
Variations in accelerometer wear (source: authors)

Adaptations on recommended standard belt wear (around the waist and sitting on the right hip) have included participants wearing accelerometers on wrists or around ankles. For wheelchair-user participants, belt wear could be uncomfortable. Figure 2 below shows some of the adaptations made by participants.

For the final 15 particpants recruited data collection time was reduced from seven days to one day of the participant’s choosing. Bearing the substantial participant burden in mind, initial analyses of the accelerometer data revealed little value in retaining a seven day protocol. Group analyses were not possible due to the variability in monitor location, and the significant variability in motor function (meaning that consistent activity intensity thresholds could not be employed). As is regularly observed in general population studies with youth [24, 25], variability in wear times also existed, further limiting the ability to generate descriptive statistics for comparison with other individuals and groups. The value of the accelerometer data was evidenced when considering a day’s activity alongside the travel diary data. A graphical representation of activity patterns highlighted times of the day where participants were more or less active, and this could be used as a point of departure for discussions around activities, settings, and participation over a given day.

### GPS

Geographic Positioning Systems (QStarz BT-Q1000XT GPS units) were worn in conjunction with accelerometers to assess the extent of young people’s spatial mobility by recording the location of the participant every 30 s. For the first 20 participants recruited these were downloaded each week-day using Q Travel, overlaid on a Google map, and displayed on a screen for mobility and hearing impaired participants and some less visually-impaired participants. Viewing the GPS tracks with participants was another way to elicit conversation about destinations visited, mobility modes used, and experiences while out and about.

**Fig. 3:**
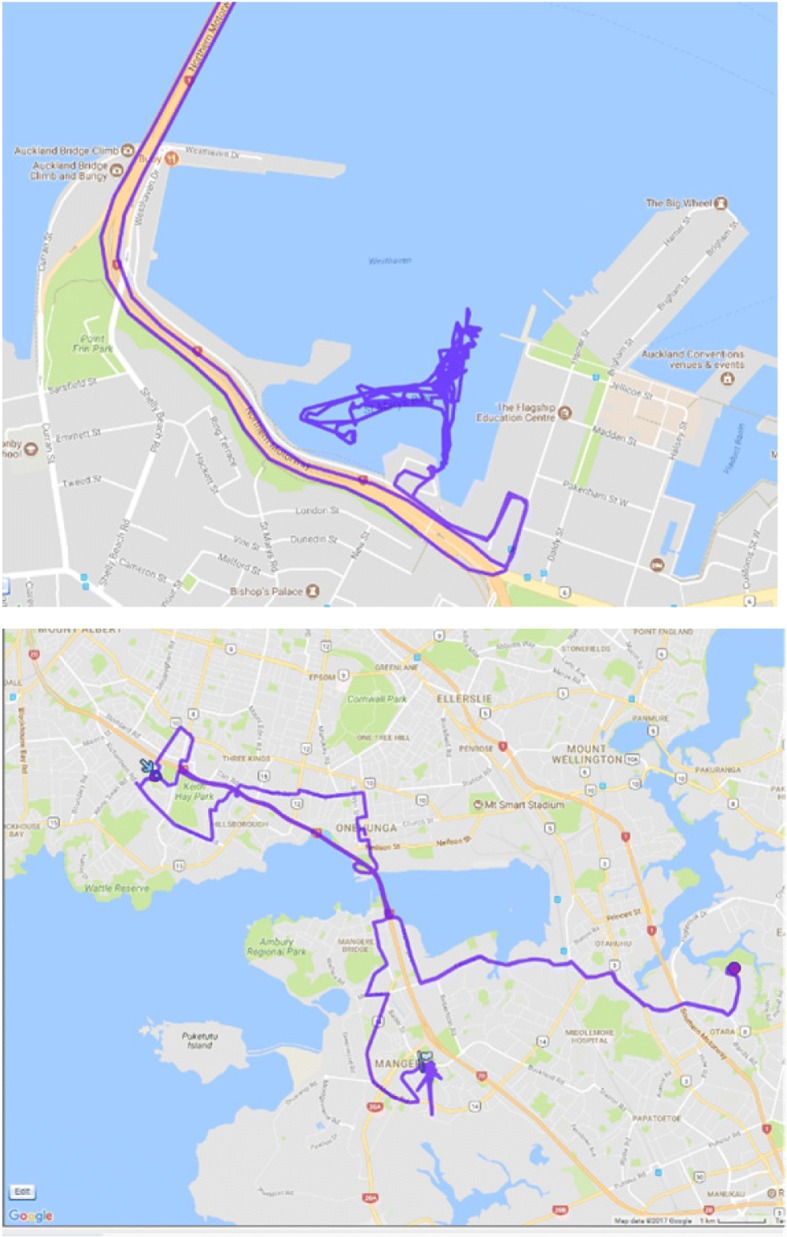
GPS tracks of two participants (source: authors)

Figure 3 (below) shows examples of GPS tracks of two mobility-impaired young people.The left hand map shows a recreational trip by car to go sailing and the right hand map illustrates a day that included visits to several destinations by public transport.

#### Modifications

Because of logistics and time constraints, for the first 20 participants GPS tracks were sometimes downloaded and discussed only every second or third day with the participants. For many vision-impaired participants who were unable to see them, tracks were downloaded after the end of the data collection session. Subsequently, as with accelerometer wear, GPS data collection was reduced to one day – a day selected by the disabled young person that illustrated community participation.

### CAPE

The CAPE (Children’s Assessment of Participation Enjoyment) is a self-report measure of the diversity, enjoyment and intensity of young people’s participation, designed for use with 6–21-year-olds. It covers 55 out-of-school activities, formal and informal, grouped in five domains: recreational, active physical, social, skill-based, and self-improvement [26]. Test retest reliability scores range from 0.72 to 0.81 for activity intensity scores and the instrument has established content and construct validity [27, 28]. Difficulty of participation is not assessed by CAPE [28]. Nevertheless it has been used widely in studies with young people with disabilities of varying types and severity [29].

The CAPE questionnaire was administered in conjunction with the Preferences for Activities with Children scale (PAC)(King et al., 2004) in various settings, at various points in the data collection process, depending on participant time constraints and availability.

#### Modifications

The CAPE was modified to take account of technological changes in forms of communication, NZ-specific activities and the inclusion of activities meaningful to older study participants (identified in collaboration with the advisory group of disabled young people). The original 55 items were extended to 62, with the addition of: going to a park; going to beach; chat/write on social networking sites or photo blogging; text messaging SMS; going out to eat; going to youth clubs or night clubs; and self- pampering/grooming (eg manicure, haircut, massage). The scoring was also simplified (e.g. by reducing response options from five to three) to facilitate its use with hearing and vision impaired participants.

The CAPE and PAC were administered in tandem. Individual items on the CAPE often became points of departure for in-depth discussion with young people on their aspirations and facilitators and barriers of participation. Thus administering the CAPE/PAC often occurred over two sessions and in combination with the qualitative in-depth interview.

### Qualitative interviews

**In-situ interview (young people):** This semi-structured interview followed and built on information gathered during quantitative data collection sessions. Topics covered included participation, sense of belonging, inclusion/exclusion, friendships, barriers to participation, hopes and fears for the future. Interview settings varied and included the young person’s home, educational facilities, cafés, and work-places. Where appropriate NZSL interpreters and AAC were used. All interviews were recorded and transcribed.

#### Modifications

The in-depth in-situ interview often spanned two or more sessions with researchers and was sometimes conducted in conjunction with CAPE/PAC data collection.

### Go along interview

Go-along interviews, originally conceived of as ‘go-along neighbourhood walking interviews’ [30, 31], followed on from the in-situ interviews, further investigating barriers/opportunities for participation. The young person and researcher took part in a conversational interview as they moved around in an outdoor locality of the young person’s choice. Go-along interviews took place in a variety of settings, including streets, parks and beaches, gyms, and often included public transport settings. The participant’s use of and experiences of the public realm were the focus of the interview as well as their responses to accessibility barriers and opportunities and interactions with others as they arose.

#### Modifications

Rather than being focused on the young person’s ‘neighbourhood’ perceptions, the go-along interviews took place in any setting the disabled young person frequented and wished to take the researcher. For vision-impaired and mobility impaired participants, it was sometimes too distracting to talk while walking – they needed to focus on their safe mobility; for hearing impaired, it was too ‘clunky’ to walk and talk through a sign language interpreter. In these cases, what worked was walking, stopping to talk, then walking on again.

### Parent interviews

In-depth interviews were conducted with parents of young people to access their understandings of characteristics of social and physical environments which constrained or facilitated young people’s mobility, participation, sense of belonging and inclusion. Demographic data and a historical narrative of their ‘journey’ with their disabled young person were also gathered. Parent interviews were variously conducted face-to-face and by telephone.

#### Modifications

Some older young people did not wish us to interview their parents and this was respected. In two instances they agreed to their parents being interviewed – but only if they were present.

### Key informant interviews

Thirteen key informant interviews were conducted with a range of people working in the disability sector: four worked with deaf and hearing-impaired young people, three with blind and low vision young people, and three with mobility impaired young people; and the remaining three key informants represented disability service providers. These interviews provided an overview of the sector and participation opportunities for disabled young people. They also supported recruitment.

### Analyses

Thematic and discursive analyses of narratives from young people and parent interview data will be undertaken. Interview data will be analysed as a single dataset and by type of impairment. Trip diary, GPS, accelerometer, and CAPE data, will provide triangulation of findings at the indivudal level to provide a comprehensive understanding of disabled young people’s participation and the constraints and facilitators of their participation.

#### Modifications

Initial objectives of investigating statistical differences and patterns in the participation, mobility and physical activity of particpants (by age, sex, and disability status) have been dropped due to wide variability in participant data. Rather than group analyses of these data they will be analysed in conjunction with qualitative data to enable a more in-depth consideration of participant experiences at an individual level.

### Workshops

Workshops will be facilitated for interested young people and parents/caregivers after initial data analyses, to discuss findings and identify strategies to increase opportunities for participation and community mobility. In addition, the efficacy of the various methods used in the study – including adaptations – will be assessed. A participant subgroup will be formed to develop a presentation to Auckland Council and other stakeholders, and to support wider dissemination.

## Discussion

The overarching aim of this study is to foreground the voices of disabled young people to identify and promote pathways for environmental change to increase opportunities for their effective community participation and full citizenship. As we engaged with the disability sector and disabled young people themselves it became evident that a flexible research process, a toolbox of research methods and a readiness to adapt research methods in response to individual capabilities/impairments, was essential if many of those recruited to the study were to be able to fully participate. To do otherwise would have been inconsistent with the study’s aims. Flexibility was also needed to ensure the ongoing support of staff in educational settings. For example, to accommodate changes in individual, classroom or school schedules, additional school visits were often needed to complete data collection.

Adopting a flexibile approach to methodology and methods meant variations to the study’s institutional ethics approval were required. Also, working with vision, hearing and mobility impaired young people necessitated nuanced differences in the uses of disability-related language between groups. On the recommendation of sector stakeholders, changes were made to tailor the language used in study information sheets to reflect current usage in NZ by the respective disability communities.

As indicated earlier, after data collection was completed for the first 20 participants, the research team reviewed the data gathered. Data collection with disabled young people is more taxing and time consuming for researchers and participants alike than comparable data collection with non-disabled young people. Aware that the young people’s impairments influenced how accelerometer data were being gathered and how GPS data were being used (or not), we questioned whether the quality and variability of data gathered warranted the intensity of research input – for participant and researcher. The multiple visits built trust between researcher and participant which facilitated the depth of qualitative data collected. However, a review of the GPS data revealed that unlike in the *Kids in the City* study, participants spent little time in their home neighbourhood. Instead the participants spent time at home (with some going nowhere), school, and at regular scheduled activities often requiring car travel across the city. Furthermore, overnight charging of the GPS units was sometimes challenging, especially for participants with vision impairment. Reviewing the accelerometer data it was also concluded there was little value in maintaining a seven-day data collection protocol. The substantial variability in unit wear times, motor ability, and monitor placement made it inappropriate to generate quantitative descriptive statistics for comparison with other individuals or groups. As such, the rationale for collecting these quantitative data shifted more towards providing objective information on activity “patterns,” by triangulating accelerometer data with travel diary data (e.g., identifying times of higher or lower activity and linking this with movement patterns, activities, and settings indicated in the travel diary and other data sources).

Following this review accelerometer and GPS data collection was reduced to a single day of the particpant’s choice. The data obtained using these methods provided an additional way to elicit understandings of disabled young people’s mobility and destination experiences; another aid to gathering in-depth and diverse qualitative data on trips or events that were meaningful to particpants. The examples in Fig. 3 are illustrative. The left-hand image shows the GPS tracks of a physically disabled participant with very limited control over her movements, who chose to wear the GPS while sailing. The traces show her tracks across the Waitemata Harbour, as she skippered the adapted small boat independently. The image on the right-hand side shows the daily movements of another mobility-impaired participant whose activities and connections to family and friends meant she regularly travelled long distances on the bus and on foot – over 70 km on this particular day. This participant was also eager to see her tracks and to keep them as a record of her movements, activities and relationships.

An unexpected discovery during data collection has been the value of the CAPE and PAC to initiate conversations with young people about their interests, experiences, hopes and dreams. Developed as a tool for measuring participation, it provided a surprising window into the lifeworlds of many participants. For hearing impaired participants, where communication was through a NZSL interpreter, the structured format of CAPE with its specific examples, suited the conventions of NZSL. Also for one physically disabled participant who utilised assisted and augmented communication, the CAPE was the most straight forward of the data collection methods as she was able to indicate a ‘yes’ or ‘no’ to questions with her big toe. Our non-standard administration and adapted scoring of the instrument means we cannot analyse the data collected as intended by the scale’s developers. Nonetheless the CAPE and PAC have been valuable research instruments for exploring the participation experiences and aspirations of many study participants.

As noted earlier, ‘go-along’ interviews were adapted to take into account participant impairments and preferences, often recording a participant’s perceptions during frequent stops rather than ‘walking and talking’.

With younger participants in educational settings, more standardised quantitative data collection processes were possible, while older participants who were not in educational settings required more flexibility with methods and protocols and on-going negotiations around what would work for them. A few were unwilling to be involved in the research at all unless they could be convinced that advocacy for disabled young people was a cornerstone of the study. Often older participants did not choose to meet at home, preferring a public place (café, or other community setting). This meant less insight into their home environment, but more understanding of places in the community that they liked to visit.

Older participants have been more involved in the co-construction of the data collection methods. Together we have discovered not just what works for different individuals in different settings, but also what participants were willing to do without being over-burdened or being ‘uncool’. Some did not want their parents to be interviewed – or in two cases, wanted to be present at the parent interview. Some of the older participants were uncomfortable with the proposed GPS tracking, and chose not to participate in this part of the project.

The range in the number of data-collection contacts between researchers and participants/families reflects the differing demographics and life-stages of participants and whether they entered the study during the earlier phase of 7-day or later phase of 1-day collection of GPS and accelerometer data (with greater numbers of contacts required during the earlier phase). In addition, data collection with many younger participants took place in schools or other educational settings, with shorter and more frequent visits required to fit around scheduled activities. Such time constraints were less likely with older participants, which often meant longer and fewer data-collection visits were possible. While the vast majority of contacts were face-to-face, some parent interviews were conducted by telephone.

Communication was challenging at times, both for informing participants about the study and during the various data collection procedures. This was particularly so when working with young Deaf and hearing-impaired participants, many of whom had experienced delays in access to oral and/or sign language. In some cases this delay impacted participants’ language comprehension and capacity to communicate with the research team (orally) or with an NZSL interpreter (using NZSL). For example, in an interview with “Alan”, a young Deaf participant who had experienced delayed access to language, conversation was interrupted as questions were repeated and reformulated, and as answers were checked and queried (“I hate Donald Trump” was initially interpreted as “I hate summer”, due to Alan’s developing signing skills). There were similar communication barriers when working with young Deaf and hearing-impaired participants who were recent immigrants to NZ, some of who had proficiency in international sign languages but were not yet fluent in NZSL. In research with mobility-impaired participants who used Augmentative and Alternative Communication (AAC) devices, researchers needed to adjust their approach to conversation, rapport-building and interviewing to fit with a slower conversational pace. For one AAC-user, some interview questions were emailed in advance so that she could consider and compose her reponses to questions in advance of the face-to-face meeting if she wished to.

Based on our experiences with this heterogeneous group (different ages, different impairments and levels and patterns of impairment), our toolbox of methods and flexible protocols have extended possibilities for participation in the research and data collection, producing rich triangulated data on the everyday lives of disabled young people. Our research methods and protocol have been adapted to take participants’ access needs into account, as well as variations in their time, energy and availability. Working flexibly with a range of methods and accommodations is enabling the research team to work respectfully and effectively alongside participants with diverse lived experience of disability and differing access needs and preferences.

While potential modifications and accommodations can be plotted out in advance, the details are best negotiated in vivo, on a case to case basis. Indeed, it is crucial that researchers are prepared to make responsive modifications in order to suit individual participants and that they avoid attempting to predict or intuit fixed protocols ahead of time. As one participant, “Elena”, explained, disabled young people regularly encounter others who are ‘set in their thinking of what disability looks like’ and who wrongly believe that they know a person’s capabilities and preferences on the basis of a diagnosis or initial meeting.

“you can never tell what exactly someone else is dealing with, so you know listening and not judging and actually finding out what they're dealing with, rather than what you assume they're dealing with, is really useful”

Thus, respect and awareness of paternalistic and ableist attitudes are also a crucial component of any research undertaken with disabled people.

In summary, a toolbox approach is proving vital to achieving this study’s overarching goal – to canvas the diverse voices of mobility, hearing and vision-impaired young people in NZ in order to increase opportunities for their effective community participation and full citizenship.

## Abbreviations

AAC: Augmentative and alternative communication
AUTEC: Auckland University of Technology Human Ethics Committee
CAPE: Children’s Assessment of Participation and Enjoyment
GIS: Geographic information systems
GPS: Global positioning systems
MUHEC: Massey University Human Ethics Committee
NZSL: New Zealand Sign Language
PAC: Preferences for Activities of Children
UAHPEC: University of Auckland Human Participant Ethical Committee

## Footnotes

1 The Ongoing Resourcing Scheme (ORS) has nine criteria covering five areas of student need for special education: learning, hearing, vision, physical, or language use and social communication. To meet ORS criteria, students must have: ongoing extreme or severe difficulty with any of the need areas, or moderate to high difficulty with learning, combined with very high or high needs in any two need areas
